# Wear and Tear of Tyres: A Stealthy Source of Microplastics in the Environment

**DOI:** 10.3390/ijerph14101265

**Published:** 2017-10-20

**Authors:** Pieter Jan Kole, Ansje J. Löhr, Frank G. A. J. Van Belleghem, Ad M. J. Ragas

**Affiliations:** 1 Department of Science, Faculty of Management, Science & Technology, Open University of The Netherlands, 6419 AT Heerlen, The Netherlands; PJ.Kole@studie.ou.nl (P.J.K.); Ansje.Lohr@ou.nl (A.J.L.); Frank.vanBelleghem@ou.nl (F.G.A.J.V.B.); 2 Zoology: Biodiversity and Toxicology, Centre for Environmental Sciences, Hasselt University, BE 3590 Diepenbeek, Belgium; 3 Institute of Water and Wetland Research, Faculty of Science, Radboud University Nijmegen, 6525 AJ Nijmegen, The Netherlands

**Keywords:** tyre wear and tear, microplastics, particulate matter, tyre rubber

## Abstract

Wear and tear from tyres significantly contributes to the flow of (micro-)plastics into the environment. This paper compiles the fragmented knowledge on tyre wear and tear characteristics, amounts of particles emitted, pathways in the environment, and the possible effects on humans. The estimated per capita emission ranges from 0.23 to 4.7 kg/year, with a global average of 0.81 kg/year. The emissions from car tyres (100%) are substantially higher than those of other sources of microplastics, e.g., airplane tyres (2%), artificial turf (12–50%), brake wear (8%) and road markings (5%). Emissions and pathways depend on local factors like road type or sewage systems. The relative contribution of tyre wear and tear to the total global amount of plastics ending up in our oceans is estimated to be 5–10%. In air, 3–7% of the particulate matter (PM_2.5_) is estimated to consist of tyre wear and tear, indicating that it may contribute to the global health burden of air pollution which has been projected by the World Health Organization (WHO) at 3 million deaths in 2012. The wear and tear also enters our food chain, but further research is needed to assess human health risks. It is concluded here that tyre wear and tear is a stealthy source of microplastics in our environment, which can only be addressed effectively if awareness increases, knowledge gaps on quantities and effects are being closed, and creative technical solutions are being sought. This requires a global effort from all stakeholders; consumers, regulators, industry and researchers alike.

## 1. Introduction

The global production of thermoplastics has grown rapidly since the start of its large-scale production around the 1950s, reaching 322 million tonnes/year in 2015 [1]. The different varieties of polymers produced have unique characteristics when compared to traditional materials, in particular in terms of durability, production costs, weight, strength, flexibility and limited electric conductivity. As a result, plastics are used increasingly in many sectors such as construction, transportation, household goods and packaging. Nowadays, the market of thermoplastics is dominated by four main classes of plastics, being polyethylene (PE; 73 million tonnes in 2010), polyethylene terephthalate (PET; 53 million tonnes in 2010), polypropylene (PP; 50 million tonnes in 2010) and polyvinyl chloride (PVC; 35 million tonnes in 2010) [2]. Besides thermoplastics, rubber is also considered a class of plastic. The 26.9 million tonnes rubber market sells two main classes: natural rubber (12.3 million tonnes in 2016) and synthetic rubber (14.6 million tonnes in 2016) [3].

As a result of the growing production of plastics, their widespread use and the mismanagement of waste, the amount of plastics in the environment is increasing rapidly. It has been estimated that between 4.8 and 12.7 million metric tonnes of plastic ended up in the ocean in the year 2010 [4]. Even on the beaches of remote areas such as Henderson Island, an uninhabited island in the South Pacific, large amounts of plastics have been detected [5]. Pollution of the environment with plastics is recognized as a serious global threat because it can negatively affect human health, aquatic organisms, as well as the economy [2,6,7,8].

Plastics end up in the ocean either as large pieces, macroplastics, microplastics (≤5 mm) or nanoplastics (≤100 nm) [9]. The sources of both macroplastics and microplastics are many and diverse. However, the implications for ecological and human health and the impact on our economy are still unknown. More research is needed to pinpoint these sources in order to enable the identification and implementation of cost-effective measures to reduce plastic pollution sources.

In this paper, the whole family of synthetic polymers, including modified natural bio-polymers, are considered to be a potential source of pollution. From an environmental point of view, thermoplastics, thermosets and elastomers all are potential sources of microplastics [9].

Car tyres release wear particles through mechanical abrasion. Several studies have suggested that wear and tear from car tyres is an important source of microplastics in the environment [10,11,12,13]. However, many questions remain. Which factors determine the release of wear and tear from car tyres, and how much is actually being released? What is the fate of these particles, once released into the environment? What impacts do the particles have on human health and on aquatic ecosystems? And, how can the emission of wear and tear from car tyres be reduced effectively? Although some of these issues have been addressed in specific scientific studies, the available knowledge is largely fragmented and often localized.

The aim of the present review is to bring together the fragmented knowledge on wear and tear of car tyres emitted into the environment and provide a global assessment of the implications for human health of this emerging source of microplastics. The review: (1) describes the characteristics of the tyre and its wear and tear; (2) summarises the amount released into the environment in different countries; (3) describes the different pathways of the wear and tear into the environment; (4) presents an estimate of the total amount of tyre wear and tear to total emissions of microplastics to the oceans; (5) discusses possible effects on humans, and (6) evaluates mitigation options. The numbers calculated in the present paper are rough estimates and should be considered as such. These numbers have been produced in a first attempt to explore the extent of the problem.

## 2. Emissions

### 2.1. Tyres

When driving a vehicle, particles are being released into the environment from its tyres. This section summarises the available knowledge on the composition of tyres, the particle generation process and the sizes of the particles generated.

#### 2.1.1. Tyre Composition

Tyres were initially only made of natural rubber, often derived from the Brazilian rubber tree (*Hevea brasiliensis*). Nowadays, a mixture of natural and synthetic rubbers is being used. Synthetic rubbers are polymers made from petroleum. About 1–4% of sulphur is added in order to vulcanise the rubber compounds, transforming them into highly elastic material, in which 1% zinc oxide serves as a catalyst. Furthermore, 22–40% carbon black is added as a filler and to make the tyre UV-resistant. In recent years, carbon is sometimes partially replaced by silica (nanoscale glass balls) [14]. Silica reduces the road resistance but it’s more difficult to form a proper bond to the rubber. In a final stage, oil is added to make the tyre less stiff and to improve its wet grip performance. Traditionally, the oil used is aromatic because of its low price and its compatibility with rubber.

The specific gravity of a microplastic influences the floating ability of the particle in water [15]. According to the United States (US) Federal Highway Administration [16], the specific gravity of tyre rubber is approximately 1.15 [16]. Banerjee and colleagues [17] mention a specific gravity of 1.17, while Dumne [18] mentions a specific gravity of 1.18. The average density of ocean waters at the surface is 1.025.

#### 2.1.2. Particle Generation

The release of wear and tear from tyres results from the contact between the road surface and the tyre. The amount and size of the particles released depend on factors such as climate (temperature), composition and structure of the tyre, the road surface, driving speed and style and the nature of the contact (e.g., rolling versus slipping) [19].

The contact between tyre and road surface causes shear and heat in the tyre; both of these processes result in the generation of wear particles. Shear forces result in the emission of comparatively large tyre particles. Heat accumulates, creating hot spots on the tyre’s surface, reaching temperatures that cause the volatile content to evaporate, which results in the subsequent release of relatively small, submicrometer, particles. Additional to the tyre wear and tear, the shear forces and the heat in the rubber also cause road wear particles to stick to the rubber wear and tear. Some researchers report that most tyre wear and tear are conglomerates with road wear [20,21].

#### 2.1.3. Size of Wear and Tear

The tyre wear and tear particle size (distribution) is dependent on many factors such as type of pavement, temperature, speed, age and composition of the tyre. However, the particles sizes reported in any particular experimental study also depend on the experimental setup.

Particles can be collected while driving a car on the road or in laboratory tests with road simulators. The samples collected typically consist of a mixture of tyre particles and particles from the road or simulator surface. Airborne particles are typically drawn by suction and measured real-time, while the coarser particles are typically collected after the test run, e.g., from the contact surface or from its direct environment like the road run off. By adjusting the suction flow, a range of particle sizes can be collected. The size ranges reported in any particular study also depend on the technical specifications of the equipment and analytical techniques used.

**Figure 1:**
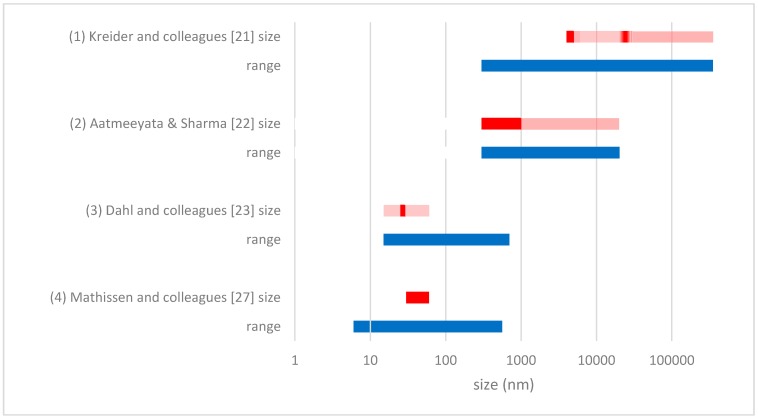
Size ranges of tyre wear and tear covered (blue bars) and detected (red bars) in four different studies (see text). Dark red suggests the size of the major number of particles [21,22,23,27].

Figure 1 provides an overview of the size ranges covered and detected in four key studies focusing on the size distribution of tyre wear and tear, highlighting the vast differences that can exist between experiments. A more extensive overview of the available studies is provided by Grigoratos and Martini [20].

Study 1 by Kreider and colleagues [21] was on the size of wear and tear of car tyres and the interaction with pavement in a road simulator using asphalt concrete pavement, i.e., a mixture of sand, gravel, crushed stone and recycled concrete bound together with asphalt. Their sampling device, consisting of a suction system located close to the tyre’s contact surface, only collected particles >0.3 μm; an upper size limit was not specified. They found particles sizes ranging from 4 to 350 μm with most particles having a size around 5 μm and 25 μm.

Study 2 by Aatmeeyata and colleagues [22] was on wear and tear on a specially constructed road simulator using concrete pavement, i.e., a mixture of sand and granite stone bound together with cement. The air was withdrawn by suction and continuously analysed by a particle size analyser on particle number and size within a 0.3 to 20 μm range. Samples were also taken from the walls and the equipment afterwards, which were considered to represent run-off material. The emission of particulate matter with particles of 10 μm or less (PM_10_) to ambient air was compared to the total weight of the run-off of particles, and was found to be less than 0.1% by weight. Almost 50% of the PM_10_ mass had a size between 0.3 and 1 μm. No size distribution was given on the course particles, i.e., particles >PM_10_.

In study 3 Dahl and colleagues [23] tested tyres in a road simulator of the Swedish National Road and Transport Research Institute. They focussed on fine air particles in the size range of 15–700 nm from both road and tyre wear and tear. The measured wear and tear was between 15 nm and 50 nm and had a distinct mean particle diameter of 27 nm. Based on transmission electron microscopy studies of the collected ultrafine wear and tear particles and on-line thermal treatment using a thermodesorber, they concluded that the particles consisted most likely of mineral oils from the softening filler and fragments of carbon black. Carbon black, which is added to tyres as a filler material and to make them UV resistant, is thought to form aggregates of 1–100 μm held together by Van der Waals bonds [24]. The particles detected by Dahl and colleagues [23] fall in the size ranges of the carbon black that is added to the tyres during the production process [25]. For example, Continental Carbon produces carbon black grade N120 with a primary particle size of 1–10 nm, grade N234 of 20–25 nm and grades N330, N339 and N351 of 26–30 nm [25,26].

In study 4 Mathissen and colleagues [27] measured air borne particle concentrations inside the car’s wheel housing while driving on an existing road. The car drove on asphalt concrete roads and the instrument was capable of measuring particles from 6 to 562 nm. When braking, accelerating and cornering, particles were measured with sizes between 30 and 60 nm. Normal driving did not result in an increase in particle number concentration.

Based on these four studies, it can be concluded that literature data show a considerable variation in the size distribution of tyre wear and tear particles. Interpretation of the experimental results is furthermore complicated by the use of different metrics (e.g., particle mass versus particle numbers), analytical difficulties to separate tyre from road particles, and the enormous variety in experimental conditions and analytical equipment. More research is needed to create a more univocal picture of the numbers and sizes of the particles generated under realistic driving conditions. Nonetheless, all studies show that tyre wear and tear will be a source of microplastics in the environment not to be ignored, covering the range from 10 nm to several 100 μm.

### 2.2. National Estimates on the Amount of Wear and Tear from Tyres

Two different approaches are typically used to estimate the amount of wear and tear from tyres. One approach uses emission factors per vehicle-km multiplied by the total mileage, and the other uses the number of tyres multiplied by the weight loss of these tyres during use. Tyres in Europe must be collected after use and processed by the manufacturer or importer [28]. Therefore, almost all used tyres will be handed in and, hence, the numbers are known.

We performed a literature search to collate national estimates on the amount of wear and tear from tyres, resulting in estimates for eight countries. Apart from Japan, the available studies on wear and tear are dominated by Western European countries. In Sweden, Norway, Denmark and Germany both emission estimation approaches have been used. The tyre number weight loss method has been used in the United Kingdom, Italy and Japan. In The Netherlands the emission factor per vehicle-km approach was used. To obtain a global estimate on the amount of wear and tear emitted into the environment, data on mileage and number of vehicles were gathered for countries for which national emission estimates were lacking. These data were found for China, India, Australia, the USA and Brazil. The emission factor method based on data from Japan and a number of European countries was used to estimate the national emission of tyre wear and tear in these countries. In this way, we calculated emissions from countries on all continents, except Africa, covering half the world’s population. Here, we first discuss the emissions factor per country, followed by the estimation of the total wear and tear on our planet.

#### 2.2.1. The Netherlands

Kole and colleagues [13] estimated the emissions of wear and tear from car tyres in The Netherlands (Table 1). They used emission factors per vehicle category and mileage data, provided by the institutes Deltares and TNO, The Netherlands Organisation for applied scientific research [29].

Of the Dutch motorways, 95% is paved with very open asphalt concrete, consisting of rock, sand, filler and bitumen. Contrary to standard asphalt, very open asphalt concrete has 15% to 25% hollow space and is used because of its capabilities to drain rainwater and to reduce noise. Of the wear and tear of tyres, 95% is considered to be captured in the pores of this very open asphalt concrete to remain trapped [29]. To maintain the draining, reducing and trapping capacities of the very open asphalt concrete, its pores have to be cleaned approximately twice per year. The washing water is processed and the dirt disposed properly [30]. If the total amount listed in Table 1 is corrected for the amount trapped in very open asphalt concrete on motorways, still 8768 tonnes will end up in the environment [13].

Verschoor and colleagues [31] calculated the wear and tear using the specific emission factors per vehicle-km method for urban, rural and highway roads (Table 2). The capturing of wear and tear in the pores of the very open asphalt concrete was taken into consideration. The total estimated amount of wear and tear for the three road types was 17,300 tonnes/year [31]. If we take the 95% capturing in the very open asphalt concrete into consideration, and subtract this from the amount of 17,300 tonnes/year we end up with 8900 tonnes/year that is released into the environment. The average of the estimated amounts (8768 and 8900 tonnes) is 8834 tonnes/year.

#### 2.2.2. Sweden

Magnusson and colleagues [32] estimated the wear and tear in Sweden based on emission per vehicle kilometre (Table 3). In Sweden, a special emission pathway exists; snow is taken from the streets and dumped into the waters. The snow will contain particles already present on the pavement even before precipitation started. Stockholm alone has permission to dump 800,000 m^3^ of snow annually from the streets into the waters around the city [32].

The Swedish National Chemicals Inspectorate [33] estimated the amount of wear and tear using the total weight of the tyres consumed in Sweden and 17% weight loss during use (Table 3).

On average the annual amount is (13,238 + 10,000)/2 = 11,619 tonnes. Here the two approaches, emission factors per vehicle-km multiplied by the total mileage, and number of tyres multiplied by the weight loss of these tyres during use provide similar results.

#### 2.2.3. Norway

Sundt and colleagues [10] used several methods to calculate the amount of wear and tear in Norway. First, they used data by the United Nations Economic Commission for Europe (UNECE) on tyre wear and tear per km, based on Russian research [26]. UNECE advises to use an emission factor of 0.033 g/tyre km for passenger cars and 0.178 g/tyre km for commercial vehicles. In their calculations, Sundt and colleagues [10] assumed all vehicles to have four wheels (Table 4).

Second Sundt and colleagues [10] calculated the wear and tear using the wear/km for passenger cars by Luhana and colleagues [34], who used an emission factor of 0.1 g/vehicle km [27] (Table 4).

Third Sundt and colleagues [10] estimated the amount of wear and tear in Norway using figures by Norsk Dekkretur (Norwegian Tyre Recycling) on disposed tyres, assuming tyres will wear 12.5% on average before being disposed (Table 4). Norsk Dekkretur organises the collection and recycling of disposed tyres in Norway.

The estimated emissions for Norway vary from 6560 to 9571 tonnes/year, with an average of 7884 tonnes/year from all the studies. As the outcomes calculated by emission/km and by disposed tyre weight loss are relatively tantamount, they are considered to be reliable. As mentioned in Section 2.2.2 the differences between the two calculation approaches could be used to improve the figures. Here the disposed tyres approach gives a higher wear and tear number. Contrary to the results the weight loss in Norway was assumed to be 12.5%; in Sweden 17%.

#### 2.2.4. Denmark

In Denmark Lassen and colleagues [12] used two different sources of emission factors to estimate the amount of wear and tear. First, they used the emission factors advised by the United Nations Economic Commission for Europe (UNECE) [35]; 0.033 g/km per passenger car tyre, 0.051 g/km per light commercial vehicle tyre and 0.178 g/km per other commercial vehicle tyre. Using these emission factors and considering 35,800, 7400 and 2000 million kilometres for passenger, light commercial and other commercial cars, respectively, the total emission of tyre wear and tear was estimated to be 1915 tonnes/year [12]. However, instead of being quoted by car, the UNECE data are given per tyre. After consulting Lassen we recalculated the emissions considering an average of four tyres per vehicle, as done by Sundt and colleagues [10], the total emission is 7660 tonnes/year (Table 5).

Second, Lassen and colleagues [12] also calculated the emission by passenger car using an emission factor of 0.1 g/vehicle km, taken from Luhana and colleagues [34]. In this way the total emission will be 6514 tonnes/year, see Table 5.

Lassen and colleagues [12] also calculated the amount of wear and tear by multiplying the number of tyres sold with the mass difference between new and disposed tyres. The weight loss of new tyres was estimated by several studies to vary between 10% and 15%. Considering the number of tyres completely outworn during the car’s life and assuming the car’s last set of tyres (i.e., before disposing the car) lost half of what an outworn tyre would lose, their calculations resulted in an estimate of 5400 tonnes/year.

Fauser and colleagues [36] also calculated the wear and tear by the number of consumed tyres multiplied by the average wear per tyre (Table 5).

On average the estimated emissions for Denmark vary from 7660, 6514, 5400 to 7310 tonnes/year, on average 6721 tonnes/year. Here the results of the two emission factors studies provide similar results; but the two studies on sold tyres differ: 5400 and 7310 tonnes/year. In this case further study should provide more insight.

#### 2.2.5. Germany

Hillenbrand and colleagues [37] calculated the amount of wear and tear by using an emission per vehicle kilometre. The figures they used are covering mileage for the year 2001 and vehicle numbers for the year 2002 (column 3 in Table 6) [37]. We used the emission per vehicle kilometre data from Hillenbrand and colleagues [37] to estimate the emissions for the year 2013 using data on total mileage from the German Federal Ministry of Transport (Bundesministerium für Verkehr) [38] (Table 6).

In a study commissioned by the German Federal Environment Agency (Umweltbundesamt) on sources of microplastics, Essel and colleagues [11] discuss two studies, i.e., the calculations by Hillenbrand and colleagues [37] mentioned above and the calculations by the German rubber trade association (Wirtschaftsverband der Deutschen Kautschukindustrie; WDK). The WDK calculated the total annual amount of wear and tear for all vehicle categories to be 60,000 tonnes [11]. Hillenbrand and colleagues [37] calculated 62,570 tonnes for buses, lorries and articulated lorries alone, while the WDK calculated only 17,000 tonnes for these categories. For the category “lorry”, Hillenbrand and colleagues [37] used 700 mg of wear per vehicle kilometre, whereas the WDK assumes a wear of 17,000/62,570 × 700 = 190 mg/km. This is about what UNECE advises for calculating a single tyre.

Baumann and Ismeier [39] calculated that the total wear and tear using amounts of wear and tear per tyre kilometre significantly differed from that calculated by UNECE; the wear and tear per tyre for heavy and articulated lorries is assumed to be about the same as for a passenger car (Table 7).

Comparing the results by Hillenbrand and colleagues [37], the WDK and Baumann and Ismeier [39], the large differences in tyre wear and tear for heavy vehicles are remarkable. WDK and Baumann and Ismeier [39] consider the wear and tear per vehicle km for lorries to be in the same order of magnitude as passenger cars. Considering the fact that experiments in a road simulator have shown a linear relationship between tyre load and tyre wear and tear [22], the figures by the WDK and Baumann and Ismeier [39] can be considered unconvincing. Considering this, we will only use the 125,188 tonnes/year as calculated by Hillenbrand and colleagues [37] and adapted to 2013 mileage, for further calculations.

#### 2.2.6. United Kingdom

The United Kingdom (UK) Environment Agency [40] estimated the amount of tyre wear and tear in the year 1996 by the weight of the 37 million tyres disposed that year, to be approximately 380,000 tonnes. The Agency assumed that a car tyre loses approximately 10–20% of its weight during use. Calculating the amount of wear and tear this way in the year 1996 results in 38,000–76,000 tonnes/year. The tyres from 1996 were probably different from current ones because of technological progress, technological changes and the impact of European Union (EU) legislation. Also, mileage will be different now. In 1996, the UK population was 58 million [41], in 2016 the UK population was 64 million [42]. Assuming the mileage per capita/year did not change, the emission would have grown to approximately 42,000–84,000 tonnes/year, or on average 63,000 tonnes/year.

#### 2.2.7. Italy

Milani and colleagues [43] considered a 10 kg passenger car tyre to lose about 1.5 kg before being abandoned after 50,000 km; this equals approximately 0.03 g/km. They calculated the total amount of wear in Italy to be 50,000 tonnes/year without providing their calculation on mileage and number of cars.

#### 2.2.8. Japan

Yamashita and Yamanaka [44] calculated the wear and tear from tyres in Japan. In 2012 there were 79,882,112 vehicles on the roads in Japan (Table 8). The average number of tyres per vehicle category is listed in column 3 of Table 8. They considered the mean life expectancy of tyres to be five years. The wear in these 5 years was calculated by considering new tyres to have an 8 mm tread depth and 1.6 mm when disposed. By measuring the diameter and tread width of a standard tyre for each category the loss for each tyre has been calculated by Yamashita and Yamanaka [44] and is given in column 5 of Table 8.

We recalculated the total emissions for the categories “normal vehicle”, “truck/bus” and “trailer”, as the calculated totals by Yamashita and Yamanaka [44] clearly had typos. The calculations of Yamashita and Yamanaka [44] resulted in an unlikely result of 15 kg/year wear and tear per capita/year. The original values reported by Yamashita and Yamanaka [44] have been put between brackets into the last column of Table 8. We recalculated the totals using the “number vehicles”, “tyres/vehicle” and “cm^3^/tyre” as provided by the authors.

According to these figures, in five years, a total of 1,042,442 m^3^ have been released from the tyres in Japan alone. The specific gravity of tyre rubber is approximately 1.15 [16]. This equates to an annual wear and tear of 239,762 tonnes/year.

#### 2.2.9. China

For China, no estimate on total tyre wear and tear in the literature was found. However, some relevant input data are available, which, combined with some assumptions, we translated to estimates of tyre wear and tear. The World Health Organization (WHO) provides the number of registered vehicles for the year 2013 [45]. Huo and colleagues [46] provided annual mileage for the categories “cars and 4-wheeled light vehicles”, “motorised 2- and 3-wheelers”, “heavy trucks” and “other” for the year 2009. No data was found on the amount of wear and tear per kilometre in China. Therefore, the amount of wear and tear per kilometre was taken from the UNECE; 0.033 g/km for cars, 0.051 g/km for light commercial vehicles and 0.178 g/km for commercial vehicles [35]. For 2-wheelers Aatmeeyata and colleagues [22] found 0.0035 g/km. These data are per tyre; for cars, we consider 4 wheels, except for heavy trucks where a conservative 6 wheels were assumed. As 2- and 3-wheelers are not differentiated, 2 wheels per vehicle were assumed (Table 9).

#### 2.2.10. India

For India, no estimate in the literature was found but, like in the case of China, data to calculate it was available. Again, the WHO provides the number of registered vehicles for the year 2011 [45]. The data on annual mileage for the year 2013 was taken from Baidya and Borken-Kleefeld [47] who studied mileage in India, published from the year 1999 up to 2006. They published data for “Megacities” and for “Rest of India” and we used the average of these two figures. The amount of wear and tear was taken from UNECE [35]. For the category “motorised 2- and 3-wheelers”, a conservative emission factor of 0.007 g per vehicle km for 2 wheelers as estimated by Aatmeeyata and colleagues [22] was used (Table 10).

#### 2.2.11. Australia

Milani and colleagues [43] calculated the total amount of wear in Australia to be 20,000 tonnes/year without providing their calculation details.

#### 2.2.12. USA

For the USA, we calculated the amount of wear and tear by using WHO data on the number of registered vehicles for the year 2011 [45], while the data on annual mileage for the year 2013 was taken from the US Department of Energy [48]. The wear and tear emission per vehicle kilometre was, like for China, taken from UNECE [35] and Aatmeeyata and colleagues [22] (Table 11).

Councell and colleagues [49] calculated the total amount of wear and tear in the USA by using both approaches, i.e., emission factors per vehicle-km and weight loss of abandoned tyres. They used a universal wear rate of 0.050 g/km, assuming 4 tyres for a passenger car, 6 for busses and lorries, and 18 for articulated lorries. Using mileage data from the US Federal Highway Administration but without showing the figures, they calculated the amount of wear and tear for 1999 to be 1,000,000 tonnes/year. Using the weight loss method they arrived at an estimate of 1,110,000 tonnes/year; so on average 1,055,000 tonnes/year.

According to the US Census Bureau, in 1999 the US population was 273 million people [50], in 2016 the US population was 324 million [42]. Assuming the mileage per capita/year did not change, the actual emissions would be 1,252,000 tonnes/year. The estimated emissions for the USA are 1,797,480 tonnes/year and 1,252,000 tonnes/year; on average 1,524,740 tonnes/year.

#### 2.2.13. Brazil

For Brazil, no estimate was found in the literature, but again data to calculate the amount is available. For Brazil annual mileage was found in a study by Tadano and colleagues [51], while again the WHO provides the number of registered vehicles for the year 2011 [45]. The mileage was based on different studies over the years 1994–2008. Again, the wear and tear was taken from UNECE [35] and Aatmeeyata and colleagues [22]. For Brazil, the WHO did provide a number of vehicles in a category “other”; as no mileage could be assigned, no wear and tear was calculated for this category. The results of our calculation can be found in Table 12.

### 2.3. Global per Capita Tyre Wear and Tear

In the previous paragraphs, the amount of tyre wear and tear from cars was estimated for different countries. Table 13 lists the estimates of the amount of wear and tear of car tyres per capita per year. The emission per capita is in the same order of magnitude for all countries, i.e., between 0.23 and 1.9 kg/year, but 4.7 kg/year for the USA.

India has the lowest wear and tear estimate, i.e., 0.23 kg/capita/year, while the USA has the highest, i.e., 4.7 kg/capita/year. The 20-fold difference can partly be explained by the fact that the USA has 0.82 cars per capita, while in India there are 0.13 cars per capita. So the car density in India is only 16% of that in the USA. The amount of wear and tear per vehicle in the USA is 6.8 kg/year compared to 1.8 kg/year for India, a 3.8-fold difference. Americans are leading in wear and tear emissions because they have more vehicles while they also travel longer distances per vehicle, especially with their lorries. In India and China the number of vehicles per capita can explain the low emission per capita per year. In The Netherlands, the capturing of wear and tear in the very open asphalt concrete explains the relatively low emission per capita per year. For Japan, the assumptions of a five-year tyre lifespan in the calculations, without considering mileage, could be a cause of the high emission per capita per year. For the rest of the countries the estimates are roughly the same; between 0.81 and 1.5 kg/capita/year.

In Table 13 the wear and tear for roughly half of the world’s population and 57% of the world’s vehicles has been estimated. The total amount of emitted tyre wear and tear from 1,011,411,004 vehicles was estimated to be 3,369,698 tonnes/year (Table 13). If the mileage from these 1,011,411,004 vehicles is considered representative for all the world’s 1,776,136,357 vehicles [52], the world total amount of emitted tyre wear and tear is 1,776,136,357/1,011,411,004 × 3,369,698 tonnes/year = 5,917,518 tonnes/year. This amount is enough to fill thirty-one of the world’s largest container ships, i.e., the 399 m Maersk Triple E with a deadweight tonnage of 194,153 [53]. On a global population of 7,323,187,457 people [42], the amount of emitted tyre dust per person equals 0.81 kg/year.

### 2.4. Airplane Tyres

Apart from road vehicle tyres, similar wear and tear is released from planes, diggers, shovels, bikes, conveyor belts, V-belts, etc. Here we make an educated guess for the amount of wear and tear by airplane tyres. To understand the order of magnitude of the wear and tear of airplane tyres we consider the Boeing 737-300 to be the average plane. The Boeing 737-300 has four main tyres, lasting about 295 start/landing cycles. The two nose wheels will last about 210 cycles. Each cycle the tyres wear approximately 0.05 mm [54]. The nose wheel diameter is 686 mm, its tread width 197 mm. The main wheel diameter is 1016 mm, the tread width 368 mm [55]. The surface of a nose wheel is 425,000 mm^2^ × 0.05 mm which translates to 21 g wear per start/landing cycle. The surface of a main wheel is 1,170,000 mm^2^ × 0.05 mm means 59 g wear per start/landing cycle. In total this means 2 × 21 + 4 × 59 = 278 g. On Dutch airports the total number of start/landings in 2016 was 571,000 [56]. A rough estimate on the annual wear and tear from airplanes released in The Netherlands is therefore 158 tonnes. Compared to the 8834 tonnes emitted tyre wear and tear in The Netherlands the 158 tonnes wear and tear by planes is 158/8834 or approximately 2%. Assuming the number of flights people travel is proportional with the distance they travel by car, the 2% can be used to make a rough estimate of the global wear and tear by aviation tyres.

### 2.5. Artificial Turf as a Secondary Source of Tyre Rubber to the Environment

The disposal of tyres is regulated in the EU under directive 2000/53/EC End-of life vehicles [28]. Tyres must be collected after use and processed by the manufacturer or the importer. Part of these tyres get a second life in Africa where requirements to profile depth are less strict. Another part of the disposed tyres is ground up to pieces between 0.7 and 3 mm and used as infill in artificial turfs [12]. Infill was considered as a good example of recycling until concerns arose about possible adverse health effects.

In Denmark building an artificial football field needs 100–120 tonnes of rubber infill [12]. After initial infilling, a field needs 3–5 tonnes of infill each year for maintenance. Lassen and colleagues [12] assume 1.5–2.5 tonnes of infill leave the field each year, ending up in the soil and sewers next to the field, in clothes of players etc. The extra 1.5–2.5 tonnes/year are needed to correct for compaction. There are about 254 registered artificial football fields in Denmark, meaning a release of 380–640 tonnes/year.

Apart from football fields, the ground up tyres are also used for running lanes, rubber mats used for playgrounds, rugby-tennis-and golf fields etc. Therefore, another 380–640 tonnes are estimated to enter the environment; bringing the total to 760–1280 tonnes/year [12]. Comparing to the 6524 tonnes/year from wear and tear this makes up 12–20%.

In Sweden the surface covered with artificial turfs is estimated at 6,117,600 m^2^ equalling 776 football fields of 7881 m^2^. The amount of infill can vary between 59 and 140 tonnes. About 90% of the artificial turfs is filled with ground up tyres. It is assumed 3–5 tonnes/year are lost from each 7881 m^2^ field; the same as the amount added each year. The loss of tyre granulate from artificial turfs is estimated at about 2300–3900 tonnes/year [32]. Comparing to the 13,000 tonnes/year from wear and tear this makes up 18–30%.

In The Netherlands there were 1800 artificial football fields in 2015, each containing about 120 tonnes infill [57]. Assuming the same 1.5–2.5 tonnes/year loss per field as in Denmark, the annual loss would be 2700–4500 tonnes/year. Comparing to the released average 8834 tonnes/year from wear and tear this makes up 30–50%.

The loss of infill to the environment is significant when compared to the amount of wear and tear from tyres. However, the particles are larger and their spreading can be easily prevented by changing the infill from tyre rubber to for example cork.

### 2.6. Other Plastic Emissions Related to Vehicle Transport

Vehicle use will inevitably be accompanied by brake and road wear. A rough estimate of the emitted amounts of brake and road particles, relative to the wear and tear of tyres, is stated below.

#### 2.6.1. Brakes

At the moment, apart from regenerative braking in electric vehicles, cars are stopped by pressing a brake pad against a rotating part of the wheel. During this action, both brake pad and counterpart will experience wear. Brake pads contain binders (phenol-formaldehyde resins), fibres (copper, steel, brass, potassium titanate, glass, organic material and Kevlar), fillers (barium and antimony sulphate, magnesium and chromium oxides, silicates, ground slag, stone and metal powders), lubricants (graphite, ground rubber, metallic particles, carbon black, cashew nut dust and antimony trisulphide) and abrasives (aluminium oxide, iron oxides, quartz and zircon). Counterparts can be cast iron and sometimes composites [58].

Hillenbrand and colleagues [37] estimated the annual amount of brake wear in Germany to be 12,350 tonnes/year. The estimated amount of brake wear is 11% of the estimated amount of tyre wear and tear in Germany. Grigoratos and Martini [58] reviewed brake wear particle emissions without considering Hillenbrand and colleagues [37]. They concluded that about 50% of total brake wear mass is PM_10_. The particle number distributions varied from bimodal with peaks at 10 and 40 nm up to unimodal with a peak at 1 μm. Generally, emitted particle sizes became smaller with increased braking power. The measured emission per vehicle for cars and 4-wheeled light vehicles was in the range of 3–8 mg/km PM_10_ and 2.1–5.5 mg PM_2.5_ [58]. If we consider the 3–8 mg/km PM_10_ to be half of the total emission from brakes and compare this to the 132 mg/km emission of tyre wear and tear [35], then the emission of brake wear is about 8% of the tyre wear and tear. Brake wear will exist all over the globe, but will depend on driving behaviour and the type of road surface [20].

#### 2.6.2. Road Markings

In Norway, 320 tonnes/year of road paint are used on the roads. Wear is heavy because of the use of salt and spikes in winter. Although markings are sometimes removed, it is assumed that all paint will wear and becomes part of the flow of microplastics [10]. In relation to the annual tyre wear and tear of 7040 tonnes/year, the 350 tonnes/year is 5%. The wear of road markings from Norway cannot be projected on the global scale since different conditions may apply, e.g., a substantial amount of unpaved roads, lacking road markings or the absence of spiked tyres.

### 2.7. Historic Increase of Tyre Wear and Tear

**Figure 2:**
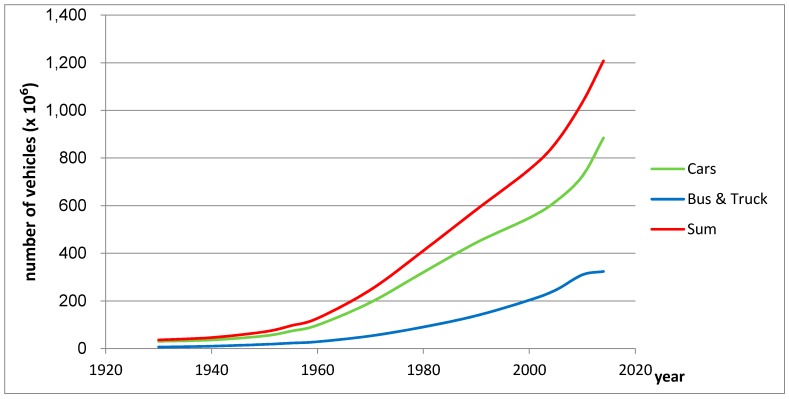
Historic increase of the global number of cars and busses and trucks [59,60,61].

To provide a basic insight on the historic figures on wear and tear of tyres, the annual growth in the world number of vehicles was used to extrapolate wear and tear figures. Possible changes in annual mileage, fleet composition and wear resistance were neglected. The US Department of Energy estimated the world’s total amount of cars, busses and trucks for the year 1950 on 70,400,000. For 2014 the same amount was estimated at 1,208,005,000 [59,60]. Walsh [61] provided the figures on the amount of cars for the years 1930 and 1940 (Figure 2).

## 3. Pathways into the Environment

**Figure 3:**
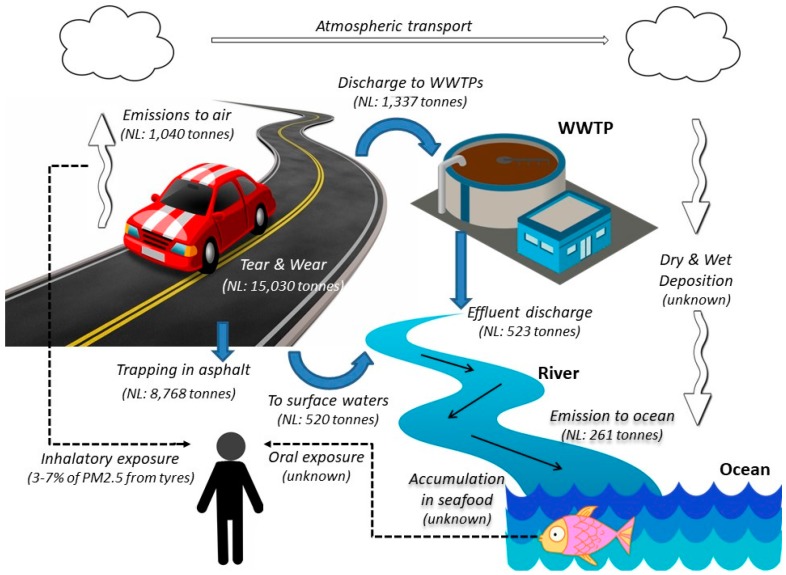
Distribution of the tyre wear and tear over the compartments. WWTP: waste water treatment plants; NL: The Netherlands.

Tyre wear and tear particles emitted on roads can be dispersed in the environment via different pathways. Small particles are typically emitted into the air and prone to air dispersal, whereas large particles will get deposited on the road surface where some parts will get trapped and other parts will be transported by rainwater runoff into soils, sewers and/or surface waters. These two most important dispersal pathways of tyre wear and tear in the environment, i.e., transport by air and by runoff, are discussed in more detail below and depicted in Figure 3.

In most studies on microplastics tyre wear and tear is not dealt with separately and we therefore use the data on microplastics as an indicator to describe the possible pathways of tyre wear and tear. None of the environmental studies in waste water treatment plants (WWTPs) or surface waters have actually identified tyre wear and tear particles, let alone its contribution to the total amount of environmental microplastics.

### 3.1. Transport by Runoff

Depending on the local situation, rainwater will flow directly into surface waters or into a sewer. In countries like Denmark and The Netherlands, two main types of sewer systems exist, i.e., combined systems leading all inflow into the WWTP, and separated systems leading rainwater directly into surface waters and just the wastewater into a WWTP. Climate change is a driver for expanding separated systems because of the expected increase and intensity of rainfall. Separated systems are built to minimise the load of relative clean rainwater to the WWTPs. Separate sewer systems have the advantage of treating undiluted wastewater, but have the disadvantage that they do not capture tyre wear and tear from runoff. For example, the length of Dutch sewers consists for 35% of separated systems and 27% of Dutch houses is connected to a separated sewer system. This implies that about 30% of the rainwater with the microplastics is discharged untreated into surface waters [62].

#### 3.1.1. Waste Water Treatment Plants

Several studies have been performed on the removal of microplastics in WWTPs (Table 14). In Sweden, Magnusson and Wahlberg [63] measured the efficiency of WWTPs with a total capacity of 1,502,000 population equivalents in the cities of Stockholm, Göteborg and Lysekil. The influent and the effluent was filtered by 20 μm and by 300 μm filters and the number of microplastics was counted by use of a microscope. On average 19.8% of the microplastics > 20 μm and 0.6% > 300 μm passed the WWTP.

In Norway, Magnusson [64] studied the WWTPs in Oslo, Tönsberg and Fuglevik, together having a capacity of 970,000 population equivalents. The method was the same as used by Magnusson and Wahlberg [63] in Sweden. On average 5.3% of the microplastics > 20 μm and 0.6% > 300 μm passed the WWTP (Table 14).

Leslie and colleagues [65] measured the efficiency of WWTPs in The Netherlands. They compared the number of microplastics in the influent and effluent of five WWTPs. The data showed that on average 28% of the microplastics between 10 μm and 5000 μm passes the WWTP.

#### 3.1.2. Amounts of Wear Particles Reaching Surface Waters

Kole and colleagues [13] combined different data on the emission and fate of tyre wear and tear from The Netherlands to arrive at an overall estimate of the amount reaching surface waters. Of the 8768 tonnes of wear and tear ending up in the environment (see Section 2), 5871 tonnes (67%) is estimated to end up in soil, 1040 tonnes (12%) in air, 520 tonnes (6%) directly in surface waters, and 1337 tonnes (15%) in sewers. From the 1337 tonnes entering sewers, 814 tonnes are estimated to remain in the WWTP and 523 tonnes to pass. So, in total 1043 tonnes, or 12% will eventually end up in surface waters [29].

Nizzetto and colleagues [66], estimated that 50% of the WWTP sludge in Europe and North America is used as a fertiliser on farmland. In European and US regulations microplastics are not named as a harmful when present in sludge to be used as fertiliser. Nizzetto and colleagues [66] used the INCA-contaminants model to study the transport of microplastics from the soil to the aquatic environment. INCA is a processed based dynamic model representation of plant/soil system dynamics and instream biogeochemical and hydrological dynamics. About 16–38% of the microplastics spread with the WWTP sludge on the land remain in the soil. Calculating with the 814 tonnes remaining in Dutch WWTP, this would imply an extra 252–342 tonnes/year will be taken by wind and rainwater to the aquatic environment. This just as an example; we do not know the Dutch percentage of WWTP sludge used as fertiliser.

#### 3.1.3. Transport by Rivers

Most microplastics will float in the water column, while lighter particles will drift on the water surface. Depending on the flow rate of the river, heavier particles may migrate along the riverbed [67]. In the river, microplastics can get covered by micro-organisms forming a biofilm that may cause the particles to sink to the riverbed [15].

Schuchardt and colleagues [68] measured microplastics concentrations in the Unterweser, a German river flowing into the North Sea. They counted 25 particles per litre in the water column, whereas 2260 particles per kg dry matter were counted in the sediment. Considering realistic river flow rates, these numbers suggest that most of the particles will remain mobile and will ultimately flow with the water into the North Sea. The Unterweser is a tidal river. The flow in the ebb stream can reach 1.4 m/s, and in the flood stream 1.2 m/s [69]. In slower flowing waters, transport might be different and heavier particles might sink to the river bed and remain in the sediment.

Nizzetto and colleagues [70] used the INCA-contaminants model to study the distribution of microplastics in the 217 km non-tidal part of the Thames from source to Teddington. Microplastics have been modelled as pure particles by their dimensions and specific mass; the formation of biofilms and the possibility of aggregation have not been incorporated in the model. They found that particles size 1–5 μm are effectively transported by the water in depended of their specific mass. Size seems to be the dominant parameter in transport by water. For sizes ≤100 μm the model predicts a retention rate ≤40% for the whole stretch.

Besselink and colleagues [15] built a model to simulate the retention of microplastics in a 40 km stretch of a small Dutch river (De Dommel) with an average flow rate of 0.2 m/s. There is a sediment settling area after 14 km and there are several weirs. The model included particle size and density, burial to sediments, aggregation to suspended solids and biofilm formation. The model was parameterized based on literature data. Aggregation was identified as the main retention mechanism for 100 nm particles, i.e., the concentration did not significantly decrease at the settling area but decreased gradually over the 40 km stretch. The simulation over the 40 km stretch showed a 60% retention rate for particles ≤1 μm and a 100% retention rate for particles ≥50 μm. Retention rate showed a minimum of 18% for 4 μm particles [15].

Combining these figures, it is possible to roughly estimate the fraction of microplastics entering surface waters to reach the ocean. According to the UNEP about 50% of the world’s population is living within 60 km from the coast [2], implying that 50% of the world’s population lives on average 30 km from the shore. This distance is quite similar to the length of the river stretch studied by Besselink and colleagues [15]. The retention rates reported by Besselink and colleagues [15] could thus be used as a starting point for estimating the average worldwide retention in rivers, especially the 60% retention reported for submicron particles (≤1 μm). For larger particles (i.e., ≥50 μm) the retention rate reported by Besselink and colleagues [15], i.e., 100%, may not be representative, since they studied a river with a relatively low flow rate, hence high sedimentation rate, and did not consider possible resuspension of sedimented particles due to extreme flooding events [2]. To cover for these processes, a 90% retention could be used as a first best guess for particles larger than 50 μm. The retention of particles in the 1–50 μm size range could then be derived by linear interpolation from the 60% retention for the submicron fraction and 90% retention for the fraction larger than 50 μm. If particle size is unspecified, an average retention of 75% seems a reasonable assumption.

### 3.2. Contribution of Tyre Wear and Tear to Plastic in the Oceans J

Jambeck and colleagues [4] estimated the amount of plastic from mismanaged waste entering from land into the oceans for almost every country. First, the amount of mismanaged waste within 50 km of the coast was estimated. Mismanaged waste is defined as either littered waste or inadequately disposed waste. Primary microplastics as tyre wear and tear are not included in these figures. Next, the percentage of plastics in the mismanaged waste was estimated. On average 11% of the mismanaged waste consists of plastics. Finally, to estimate the amount of mismanaged plastic waste entering the ocean, the San Francisco Bay watershed was studied. The unmanaged amount was compared to the amount collected by street sweeping, in storm water catchments and pump stations. The percentage of uncollected plastic waste available to enter the ocean was estimated at 61% on average with a minimum of 36% and a maximum of 95% [4].

Table 15 compares the total amount of plastics estimated to enter the ocean in Norway and The Netherlands to the amount of tyre wear and tear. In Norway, 7884 tonnes/year of tyre wear and tear is being released to the environment, including both synthetic and natural rubbers (Section 2.2.3). About half this amount, i.e., 3942 tonnes/year, is expected to end up in the ocean [10]. In The Netherlands, 1043 tonnes of wear and tear is estimated to enter surface waters every year (Section 3.1.2). Assuming an average retention of 75% (Section 3.1.3), 261 tonnes will reach the oceans. Both estimates do not include the contribution from atmospheric deposition, since insufficient data are available to estimate this source reliably.

The relative contribution of tyre wear and tear to the overall load of plastics in the oceans varies considerably between these two countries, i.e., 0.9% for The Netherlands and 31.9% for Norway. Considering The Netherlands a “best case” for emission of tyre wear and tear into the oceans, and Norway a “worst case”, the relative contribution of tyre wear and tear to the global loading of the ocean with plastics can be estimated to be in the range of 5–10%. Important uncertainties and variable factors in this estimate include (1) the amount of tyre wear and tear retained in sewers and WWTPs; (2) the river retention of tyre wear and tear; and (3) the amount of tyre wear and tear directly discharged to the ocean.

### 3.3. Transport by Air

In terms of mass, only a small fraction of the tyre wear and tear particles generated become airborne. This is because larger particles (>10 μm) tend to deposit on the road or close to it and these particles constitute the major part of the mass being released. Based on a review of the available literature, Grigoratos and Marini [20] conclude that the mass fraction becoming airborne varies between 0.1% and 10%, although some studies report fractions up to 30% [20].

The fate of airborne wear particles strongly depends on size. Distinction can be made between particles >10 μm, 1–10 μm, 0.1–1 μm and <0.1 μm. The behaviour of particles larger than 10 μm in diameter is governed by gravitational forces and will typically deposit close to the source. Hence, these particles typically constitute only a small fraction of the airborne particles. The behaviour of particles in the 1–10 μm range strongly depends on particle characteristics and local conditions. These particles can stay in the air for minutes to hours and typically travel distances varying from hundred meters to as much as 50 km. Specific transport data on the 0.1–1 μm fraction are lacking, but it is well known that PM_2.5_ particles (i.e., particles <2.5 µm) can stay in the air for days or weeks and travel more than a thousand kilometres. Particles in the nano range (i.e., <0.1 μm) are subject to various processes influencing their fate. Due to their small size, electrostatic forces may result in adsorption of nanoparticles to the road surface or vehicle carcass [71]. Furthermore, Dall’Osto and colleagues [72] demonstrated that tyre wear and tear nanoparticles may be encapsulated by road wear resulting in larger particles of mixed composition [72]. This may provide an explanation for the fact that several studies have detected nanosized tyre particles under laboratory conditions, whereas such particles are less often detected under more realistic road driving conditions. Nonetheless, a few studies also report the emission of nanosized particles under realistic road driving conditions [23,27]. These conflicting results make it difficult to assess whether and how many nanosized tyre wear and tear particles are being released to air. However, even if nanoparticles are being released their transportation range seems limited because these particles are subject to sorption and aggregation processes.

The contribution of tyre wear and tear to airborne PM_10_ has been estimated in several studies, mostly focusing on quantifying the contribution of non-exhaust PM emissions relative to exhaust PM emissions. Based on data from several European countries, Ketzel and colleagues [73] estimated that 50–85% of the total traffic PM_10_ emissions originates from non-exhaust sources [73]. The large variation is due to factors such as the degree of precipitation (i.e., resuspension is less under wet conditions), road surface characteristics and the type of tyres (i.e., studded tyres result in a substantial increase of non-exhaust PM_10_ emissions). Tyre wear and tear is typically expected to contribute least of the non-exhaust sources, i.e., resuspension, road wear, brake wear and tyre wear and tear. Estimations range from 0.1 to 10% for airborne PM_10_ and 3–7% for airborne PM_2.5_ [20]. However, one should keep in mind that the contribution of tyre wear and tear to traffic PM10 may have been underestimated in these studies due to the encapsulation of nanosized particles as reported by Dall’Osto and colleagues [72].

We were unable to identify studies that explicitly quantify the amount of tyre wear and tear that ends up in the ocean after transportation by air. Although the fraction of tyre wear and tear in airborne PM_10_ is generally considered low (i.e., <1%), deposition of marine aerosols may still contribute significantly to the overall load in our oceans since 70% of the earth’s surface is covered by oceans. The sources and composition of marine aerosols have been extensively studied, but remarkably few studies have looked at the presence of microplastics in marine aerosols, let alone tyre wear and tear. The few studies that are available show conflicting results. Dall’Osto and colleagues [72] quantified tyre dust in aerosol samples taken at different European monitoring sites, including three marine sites [72]. For each of these sites at least 100,000 particles were analysed and less than 5 tyre wear and tear particles per site were detected (Dall’Osto, personal communication, 19 July 2017) [74], implying a negligible amount of tyre dust in marine aerosol. Fu and colleagues [75] analysed the organic molecular composition of marine aerosol samples collected during at the Arctic Ocean. They analysed more than 110 individual organic compounds which were grouped into different classes based on the functionality and sources. One class was labelled “plastic emission” based on the detection of phthalate esters. This group was reported to be the fourth in terms of source strength with a mean relative abundance of 8.3%. Although tyre wear and tear by no means is the only source of phthalates esters [76], these results suggest that airborne plastic particles can be transported over long distances and may ultimately be deposited into our oceans.

Unfortunately, data are lacking to reliably estimate the fraction of airborne tyre wear and tear ultimately reaching our oceans. One reason is the complete lack of data on the (photo)degradation of wear and tear particles in ambient air. It is therefore recommended to perform more research on the degradation of tyre wear and tear particles in air and the presence of tyre wear and tear in marine aerosols, e.g., by focussing on the use of distinct tracers such as hydrogenated resin acids and benzothiazoles [77,78].

## 4. Health Effects

Ultimately, humans and ecosystems can be exposed to the tyre wear and tear released into the environment. For humans, the most relevant exposure route is inhalation of airborne particles [20,79] Marine and other aquatic organisms may be exposed to tyre wear and tear through ventilation (gills) and feeding [80]. Filter feeders and sediment dwelling organisms can be expected to have the highest exposure because their feeding strategy involves the direct uptake of food particles from the water and/or sediment. Many of these organisms, e.g., mussels and oysters, are important commercial seafood species. Hence, the question arises whether human health may be at risk due to the consumption of polluted seafood. These human health issues are discussed in more detail below.

### 4.1. Health Effects from Inhalation

It is well-known that inhalation exposure to airborne particles can trigger a wide range of adverse health effects [81]. The effects depend on factors such as the particle concentration in the air, the size distribution of the particles, their shapes, their chemical composition and ventilation intensity. From a mechanistic viewpoint, distinction is often made between physical effects of particles (i.e., resulting from the physical interaction between particle and tissue) and toxicological effects of particle leachates. This distinction is not always easily maintained for tyre wear and tear particles since the samples collected and tested in practice often represent a heterogeneous mixture of many different chemicals and structures, including rubber, synthetic polymers, Zn, carbon, other additives, road wear, brake wear and exhaust [72]. Here, we first briefly review the available literature on the physical effects of plastic particles, before discussing the toxicity of leachates from tyre wear and tear. We then review the available toxicity studies with tyre wear and tear and discuss its toxic potential based on its contribution to PM_2.5_ and the global health burden attributed to air pollution.

Larger particles (>1–10 μm) penetrate less deep in the lung and are more likely to be subject to mucociliary clearance [82]. Particles <1 μm can get deposited deeper in the lung and for these particles uptake across the epithelium is possible, e.g., by means of diffusion, passive cellular penetration or active uptake (endocytosis) [83]. Studies with model mammalian systems suggest that submicron particles can translocate to the lymphatic and circulatory systems, but it is not yet clear to what extent this phenomenon results in accumulation in secondary organs and poses a threat to the immune system or cell health [84].

An increase in respiratory disorders after exposure to airborne plastic particles has been reported in several occupational studies, i.e., workers processing nylon flock, different types of plastic fibres and synthetic textile [83]. Effects detected include respiratory irritation, reduced lung capacity, coughing and increased phlegm production. This is in line with findings of histopathological analyses of lung biopsies reporting interstitial fibrosis and locations of inflammatory lesions. Although no evidence for increased lung cancer was found in nylon flock workers, slightly higher levels of plastic microfibers have been detected in malignant lung tissue taken from patients with different types of lung cancer than in nonneoplastic lung tissue [83].

Besides effects resulting from the physical contact between particles and cells or tissues, effects may also be triggered by chemicals leaching from wear and tear particles. Several studies have shown that toxic effects are associated with the metals in these particles. For example, Gottipolu and colleagues [85] found that the water-soluble zinc and copper fraction of tyre dust was associated with increased levels of cardiac oxidative stress detected in rats exposed to high levels of this dust (5 mg/kg rat) [85]. The presence of zinc has also been associated to the toxicity of tyre particles leachates in studies with human lung cells [86]. Findings of epidemiological studies seem to confirm that airborne Zn particles can trigger acute respiratory responses [87]. The toxic potential of organic components in tyre wear and tear has been demonstrated in human lung cells [86].

In vitro tests in which human lung cells and macrophages were exposed to tyre wear and tear particles have reported inflammatory responses, e.g., secretion of interleukin-6, interleukin-8, tumour necrosis-factor α and altered protein levels [88,89]. In vivo tests in which animals were exposed to samples containing tyre wear and tear show contradictory results. A study in which adverse effects were detected in rats exposed to air PM collected at locations with high traffic density, related these effects to different sources, one of these being tyre wear and tear (Zn) [90]. However, other studies found that tyre and road wear particles generated in a road simulator laboratory triggered only minimal lung alterations, considered insufficient in extent or severity to have an impact of pulmonary function [91,92,93].

It can be concluded that unambiguous toxicological data on the inhalatory effects of tyre wear and tear particles are currently lacking. However, tyre wear and tear has been estimated to contribute 3–7% to PM_2.5_ (see Section 3.3) and the toxic potential of PM_2.5_ has been well established. Using PM_2.5_ as an exposure metric, the World Health Organisation recently estimated that outdoor air pollution was responsible for 3 million deaths globally in the year 2012 [94]. This suggests that tyre wear and tear may contribute to the global health burden due to air pollution. However, unambiguous conclusions cannot be drawn since is not yet known what components in PM_2.5_ contribute most to its detrimental effects. This stresses the urgency of identifying those components.

### 4.2. Health Effects from Food Intake

To what extent foodborne exposure to microplastics poses a human health risk is not well-understood [95]. The risk depends on the one hand on the level of exposure and on the other on the inherent toxic potential of the wear and tear particles. Since uptake of microplastics has been documented for hundreds of aquatic food species at several trophic levels (see [96] and the references therein), also ingestion of tyre wear and tear is to be expected. As a consequence, even though the presence of tyre wear and tear in aquatic food has not been documented yet, human exposure to microsized and nanosized tyre particles via the consumption of aquatic food species seems apparent, in particular in case of aquatic animals that are consumed whole. Due to atmospheric deposition, microplastics can also enter terrestrial systems and the soil [97]. For instance, synthetic fibres and fragments have been identified in honey [98]. It can therefore be assumed that tyre wear and tear can also end up in terrestrial food.

Potential toxic effects of tyre wear and tear particles via the food can either be local or systemic. Systemic effects depend on intestinal absorption followed translocation to the target organ or site. Currently, there are no specific studies on the intestinal uptake of tyre wear and tear, but several intestinal uptake mechanisms are documented for microsized and nanosized particles [99,100,101]. In fact, nanosized particles with a diameter below 200 nm could display a higher bioavailability due to the potential uptake via receptor-mediated endocytosis [102]. However, bioavailability is influenced by the interaction with proteins and other biomolecules, leading to the formation of a biomolecule corona, a process which in turn is affected by gastrointestinal digestion processes [103]. Another important factor is the interaction with mucus, the intestinal wall is known to provide an effective barrier against micro- and nanoparticles as the majority is discarded from the intestine or trapped in mucus before reaching the epithelium [104]. Yet, topical application of particles such as polystyrene and diesel particulates is shown to disrupt the mucus barrier which could possibly increase bioavailability [105]. Nevertheless, in general it can be assumed that in analogy with plastic particles, tyre wear and tear of different size classes can indeed be internalized by the intestinal epithelium, but the effective uptake is probably low [99]. Tyre wear and tear particles are expected to cause local inflammatory effects in the intestinal lumen, in a similar way as observed under respiratory exposure in mice [101,106]. In addition, the presence of microsized (tyre) particles in the intestinal lumen could also pose a threat due to the potential leaching of toxic substances to the intestinal tissue. Zinc oxide, the main form of zinc in tyre wear and tear, is considered relatively non-toxic, but several compounds could pose a threat such as the carcinogenic polycyclic aromatic hydrocarbons (International Agency for Research on Cancer (IARC) Group 1–3), carbon black (possibly carcinogenic, IARC Group 2B), non-redox-active heavy metals like cadmium, lead, nickel and redox-active metals like copper and iron [107,108]. In fact, toxic effects from aqueous leachates of tyre wear and tear have been documented in the green alga *Raphidocelis subcapitata*, the water flea *Daphnia magna* and the frog embryo *Xenopus laevis* [109,110] and exposure to organic tyre wear and tear extracts led to genotoxic effects due to oxidative stress in human lung cell line A549 [86]. Although these results clearly suggest a potential risk, extensive toxicological research is necessary to enable a comprehensive human effect assessment.

## 5. Mitigation

### 5.1. Wear Resistant Tyres

The European Tyre Labelling Regulation 1222/2009/EC [111] requires the labelling of tyres for rolling resistance (aiming at lower fuel consumption), wet slip resistance (aiming at improved safety), and noise (aiming at noise reduction). Wear and tear of tyres is not explicitly covered by a European regulation. The introduction of new regulations aiming at the reduction of wear and tear could thus be an option. However, it should be realized that the different regulatory requirements imposed on tyres are interrelated. Within the tyre technology, this is known as the “magic triangle”, i.e., the relationship between rolling resistance, slip resistance and wear resistance. Improving one, will deteriorate the other (Noordermeer, personal communication, 17 May 2017) [112]. An improved wear resistance would thus result in a poorer rolling resistance and slip resistance. This means that a compromise has to be sought between fuel consumption (through rolling resistance), safety (slip resistance), durability (wear resistance) and environmental considerations (wear resistance). On one hand this requires the application of techniques that can reflect these different dimensions in a common denominator, e.g., Life Cycle Impact Assessment (LCIA), and on the other hand the involvement of different stakeholders (e.g., tyre industry, government, environmentalists, etc.) to weigh the different values involved, e.g., involving the application of Multi-Criteria Analysis (MCA).

### 5.2. Electric Cars

**Figure 4:**
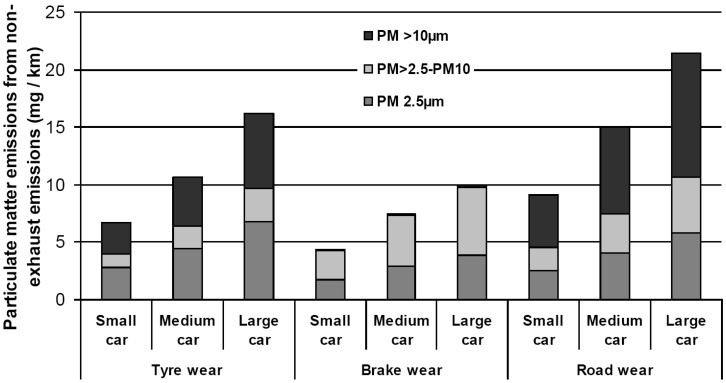
Non-exhaust particulate matter (PM) emissions by source and car size, from Simons [113] based on Ntziachristos and Boulter [117].

Experiments with motorbike tyres in a road simulator showed a linear relationship between tyre load and tyre wear and tear [22,113] see Figure 4. As electric cars (E-cars) are, due to their battery pack, heavier than Internal Combustion Engine (ICE) cars, E-cars will produce more tyre wear and tear [113,114].

Table 16 compares the weights of some common ICE-cars and their electric alternatives. On average, the electric versions are approximately 20% heavier. Assuming a linear relationship between weight and tyre wear and tear emission, this emission will be about 20% higher for E-cars. It can be concluded that current electric cars will not solve the particulate matter problem. They will reduce the PM_10_ problem by eliminating exhaust emissions and reduced brake wear [115], but at the same time they will increase the problem by increased emission of tyre wear and tear. Only if the weight of batteries is substantially reduced, which seems to be likely in the near future [116], a net gain in terms of human health effects seems evident.

### 5.3. Self-Driving Cars

Self-driving cars can be programmed to reduce wear and tear. Examples include quiet acceleration, taking bends slowly and improved anticipation to traffic circumstances resulting in fewer intense braking events. If all cars would be computer driven, driving could also become more intrinsically safe. This could alter the balance of the magic triangle between rolling resistance, slipping resistance and wear resistance (see Section 5.1), resulting in a higher priority for wear resistance and thus less wear and tear. After all, road safety of self-driving cars will be part of the Internet of Things (IoT) they will rely for their safety more on Artificial Intelligence (AI) (i.e., controlled acceleration and braking; anticipation of traffic circumstances, being interconnected) than on the slipping resistance of tyres.

### 5.4. Sewers and Waste Water Treatment Plant Efficiency

Sewers and WWTPs play an important role in the loading of surface waters (see Section 3.1). One option to reduce this loading is to increase WWTP treatment efficiency. However, to our knowledge no studies are available that systematically analysed the processes responsible for removing wear and tear particles, or microplastics in general, from WWTPs. It seems plausible that sedimentation plays an important role for larger particles, and potentially also for smaller particles after aggregation. Like demonstrated for graphene nanomaterials, the addition of a suitable coagulant may reduce the particles from the wastewater stream [118]. The application of tertiary treatment techniques such as UV radiation and oxidation techniques are also likely to remove wear and tear particles from the water phase. Herbort and Schuhen [119] proposed the application of innovative inorganic-organic hybrid silica gels which have the ability to remove stressors such as microplastics from wastewater.

A second option to reduce wear and tear loading of surface waters is to limit the use of separated sewer systems in which the runoff from roads is discharged directly into surface waters. However, this would require a substantial increase of WWTP capacity and thus be expensive. A more viable option is to develop a more efficient trapping device for wear and tear particles to be applied before the runoff enters the sewer system (e.g., in the gutter) or before it is discharged into surface water. Collection of runoff and temporary storage in a sedimentation basin could already substantially reduce the load, particularly for the larger particles.

### 5.5. Open Asphalt Concrete

The pavement material is an important factor in tyre wear and tear. The pavement could be designed to reduce wear [120]. Designing roads using (very) open asphalt concrete could reduce emissions while catching the coarse part of tyre wear and tear [13].

## 6. Conclusions

The present review shows that wear and tear from tyres constitutes a significant global source of microplastics in the environment. The emission of tyre wear and tear from cars was estimated for different countries using two different methods, i.e., using (1) emission factors per vehicle-km and total mileage; and (2) the number of tyres used combined with their weight loss. Both methods resulted in comparable results, forming an indication that emissions can be reliably estimated with either of both methods. The emission per capita is in the same order of magnitude for all countries, i.e., between 0.23 and 1.9 kg/year, with a 4.7 kg/year outlier for the USA.

Although quantification of environmental pathways remains a challenge, the relative contribution of tyre wear and tear to the total global amount of plastics ending up in our oceans was roughly estimated to be in the range of 5–10%. This makes wear and tear from tyres at least as important as plastic bottles, bags and fibres released from clothing during washing [2]. These numbers underline that tyre wear and tear deserves a higher place on the political agenda and that emission reduction of tyre wear and tear should be given higher priority than it currently receives.

Although the pathways and potential adverse effects of tyre wear and tear are largely known, quantification of these pathways and the associated risks remains a tough scientific challenge. First and foremost, it remains difficult to quantify the emission of tyre wear and tear under realistic driving conditions and to characterize the particles that are being released in terms of numbers and sizes. An important complicating factor is the mixing with other particles, i.e., road and brake wear. The development and application of unique robust tracers for tyre wear and tear can substantially improve our understanding of the amount of wear and tear being released into the environment and its dispersal with ambient air and runoff [77,78]. For the route via runoff, important knowledge gaps include the amount of wear and tear trapped in road surfaces and soils, the removal efficiency in WWTPs and the fate (i.e., retention) of wear and tear particles in surface waters. For the route via air, the most important challenge is to quantify the amount of tyre wear and tear in environmental matrices far away from the source, e.g., in marine aerosols. This could confirm whether deposition of tyre wear and tear constitutes a significant source of microplastics in our oceans. For all environmental dispersal routes, quantification of degradation remains an important challenge.

In terms of human health risks, the first and foremost challenge is to quantify human exposure in a realistic manner. Numerous studies have shown that fine particles, including tyre wear and tear, can trigger a range of adverse health effects (see Section 4). However, without reliable information on realistic exposure levels it remains unclear whether such effects are likely to occur in real-life. Exposure is best characterized for the inhalation route, where it was estimated that tyre wear and tear contributes 3–7% to the ambient PM_2.5_ fraction (see Section 3.3). This suggests that tyre wear and tear may contribute to the global health burden of air pollution which has been projected by the WHO at 3 million deaths in 2012. However, this statement should be treated with care since it is not yet known what components in particulate matter contribute most to the effects. Exposure is much less well characterized for the intake of tyre wear and tear with (sea)food. Although there is no acute reason for concern, some specific target groups such as people eating large amounts of mussels, oysters and other seafood species that are consumed without removal of the intestines, may be exposed, especially when living in highly polluted coastal areas. Research on exposure should therefore primarily focus on these specific types of seafood, target groups and locations.

Industry, regulators and consumers quickly undertook action when it became clear that microbeads in cosmetics contributed to the microplastics in the environment [121]. This was relatively easy since there was a simple solution, i.e., replacing the plastic microbeads by natural beads, e.g., ground walnut shell. Tyre wear and tear constitutes a much more important source of microplastics in the environment, but awareness is low and currently there is no alternative for tyres. It can be concluded that tyre wear and tear is a stealthy source of microplastics in the environment, which can only be addressed effectively if awareness increases, knowledge gaps are being closed and creative solutions are being sought. This requires a global effort from all stakeholders; consumers, regulators, industry and researchers alike.

## Figures and Tables

**Table 1:** Calculation of the amount of tyre wear and tear in The Netherlands by Kole and colleagues [13].

	Wear mg/km	Mileage in 2012 Built-Up Area (×10^6^ km)	Mileage in 2012 Rural Roads (×10^6^ km)	Mileage in 2012 Motorways (×10^6^ km)	Total Wear 2012 tonnes/year	Corrected for 95% Trapped in Motorways
Passenger car	100	20,876	36,472	45,349	10,270	6263
Articulated lorry	495	274	867	3418	2257	762
Lorry	600	406	525	1434	1419	659
Other					1084	1084
Total					15,030	8768

**Table 2:** Calculation of the amount of tyre wear and tear for urban, rural and highway roads in The Netherlands by Verschoor and colleagues [31].

	Urban Roads			Rural Roads			Highway Roads		
	Wear mg/km	Total Mileage in 2012 (×10^6^ km)	Total Wear 2012 tonnes/year	Wear mg/km	Total Mileage in 2012 (×10^6^ km)	Total Wear 2012 tonnes/year	Wear (mg/km)	Total Mileage in 2012 (×10^6^ km)	Total Wear 2012 tonnes/year
Moped	13	1608	21	9	690	6	10	0	0
Motorcycle	60	393	24	39	1100	43	47	1089	51
Passenger car	132	20,959	2767	85	36,622	3113	104	45,541	4736
Van	159	2670	425	102	5331	544	125	8649	1081
Lorry	850	412	350	546	533	291	668	1453	971
Truck	658	277	182	423	876	371	517	3455	1786
Bus	415	354	147	267	207	55	326	82	27
Special vehicle light	159	22	3	102	44	4	125	72	9
Special vehicle heavy	850	59	50	546	76	41	668	210	140
Total		26,754	**3969**		45,479	**4468**		60,551	**8801**

**Table 3:** Calculation of the amount of tyre wear and tear in Sweden [32,33].

	Based on Annual Mileage: Magnusson and Colleagues [32]			Based on Tyres Sold: Swedish National Chemicals Inspectorate [33]		
	Wear mg/km	Total Mileage in 2012 (×10^6^ km)	Total Wear 2012 Tonnes/Year	Mass of Tyres Consumed Annually kg	Weight Loss (17%) kg	Total Wear 2002 tonnes/year
Passenger car	50	62,940	3147			
Bus/lorry	700	14,416	10,091			
Total		77,356	**13,238**	60,000,000	10,000,000	**10,000**

**Table 4:** Calculation of the amount of tyre wear and tear in Norway [10].

	Based on Annual Mileage: Sundt and Colleagues [10] Using United Nations Economic Commission for Europe (UNECE) Data			Based on Annual Mileage: Sundt and Colleagues [10] Using Data by Luhana and Colleagues [34]			Based on Disposed Tyres: Sundt and Colleagues [10]			
	Wear mg/km	Total Mileage in 2013 (×10^6^ km)	Total Wear 2013 tonnes/year	Wear mg/km	Total Mileage in 2013 (×10^6^ km)	Total Wear 2013 tonnes/year	Weight of Disposed Tyres	Times Re-Treaded	Weight Loss from New	Total Wear 2013 tonnes/year
Passenger car	132	30,000	3960	100	30,000	3000	42,000	0	12.5%	6000
Heavy transport	712	5000	3560	712	5000	3560	10,000	2.5	12.5%	3571
Total		35,000	**7520**		35,000	**6560**	52,000			**9571**

**Table 5:** Calculation of the amount of tyre wear and tear in Denmark using the wear data on passenger car [12,34,36].

	Based on Annual Mileage: Lassen and Colleagues [12] Using UNECE Data [35]			Based on Annual Mileage: Lassen and Colleagues [12] Using Data by Luhana and Colleagues [34]			Based on Tyres Sold: Fauser and Colleagues [36]		
	Wear (mg/km)	Total Mileage in 2014 (×10^6^ km)	Total Wear 2014 tonnes/year	Wear (mg/km)	Total Mileage in 2014 (×10^6^ km)	Total Wear 2014 tonnes/year	Number of Tyres Consumed Annually	Weight Loss per Tyre kg	Total Wear 1990 tonnes/year
Passenger car	132	35,800	4726	100	35,800	3580	1,900,000	2.4	4560
Light commercial	204	7400	1510	204	7400	1510			0
Commercial car	712	2000	1424	712	2000	1424	250,000	11	2750
Total		45,200	**7660**		45,200	**6514**			**7310**

**Table 6:** Calculation of the amount of tyre wear and tear in Germany [37,38].

	Wear (mg/km) [37]	Total Wear 2001/2002 tonnes/year [37]	Total Mileage in 2013 (×10^6^ km) [38]	Total Wear 2013 tonnes/year
Moped	22.5	88	4700	106
Motorcycle	45	621	12,300	689
Passenger car	90	46,017	615,100	55,359
Bus	700	2590	3300	2310
Lorry	700	43,540	64,300	45,010
Articulated lorry	1200	16,440	16,700	20,040
Other	180	2124	9300	1674
Total		111,420	725,700	125,188

**Table 7:** Calculation of the amount of tyre wear and tear in Germany by Baumann and Ismeier [39].

	Number of Vehicles in 1995	Wear per Tyre mg/km	Average Number of Tyres	Average Mileage in 1995 km	Total Wear 1995 tonnes/year
Passenger car	40,500,000	20	4	14,200	46,008
Bus	46,900	32	6	46,900	422
Lorry < 7.5 tonnes	1,961,000	36	5	25,000	8825
Lorry > 7.5 tonnes	254,000	21	9	70,000	3360
Articulated lorry	124,100	18	15	82,000	2748
Total					61,363

**Table 8:** Calculation of the amount of tyre wear and tear in Japan generated in 5 years [44].

	Number of Vehicles	Tyres/Vehicle	Total Number of Tyres	Wear and Tear in cm³/Tyre	Total Wear m³	Total Wear m³ as Reported in [44]
Motorcycle	3,402,405	2	6,806,810	1136	7733	(7733)
Light vehicle	24,756,432	4	99,025,728	1780	176,266	(176,266)
Normal vehicle	43,350,396	4	173,401,584	2880	499,397	(4,993,966)
Truck/bus	2,790,562	10	27,905,620	5484	153,034	(1,666,803)
Trailer	2,463,607	14	34,490,498	5973	206,012	(1,891,459)
Total	76,763,402				1,042,442	(8,736,183)

**Table 9:** Calculation of the amount of tyre wear and tear in China [22,35,45,46].

	Number Vehicles	Annual Mileage	Wear and Tear g/km	Wear and Tear Tonnes
Cars and 4-wheeled light vehicles	137,406,846	19,400	0.132	352,000
Motorised 2- and 3-wheelers	95,326,138	5600	0.007	3740
Heavy lorries	5,069,292	60,000	1.068	325,000
Other (light duty lorries)	12,335,936	30,000	0.204	75,500
Total	250,138,212			756,240

**Table 10:** Calculation of the amount of tyre wear and tear in India [22,35,45,47].

	Number Vehicles [45]	Annual Mileage [47]	Wear and Tear g/km [22,35]	Wear and Tear Tonnes
Cars and 4-wheeled light vehicles	38,338,015	10,275	0.132	51,998
Motorised 2- and 3-wheelers	115,419,175	6600	0.007	5332
Heavy trucks	4,056,885	50,075	1.068	216,963
Buses (light duty trucks)	1,676,503	53,745	0.204	18,381
Total	159,490,578			292,674

**Table 11:** Calculation of the amount of tyre wear and tear in the USA [22,35,45,48].

	Number Vehicles [45]	Annual Mileage km [48]	Wear and Tear g/km [22,35]	Wear and Tear Tonnes
Cars and 4-wheeled light vehicles	245,669,103	18,095	0.132	586,800
Motorised 2- and 3-wheelers	8,437,502	3899	0.007	230
Heavy lorries	10,270,693	109,685	1.068	1,203,000
Buses (light duty lorries)	666,064	54,803	0.204	7450
Total	265,043,362			1,797,480

**Table 12:** Calculation of the amount of tyre wear and tear in Brazil [22,35,45,51].

	Number Vehicles [45]	Annual Mileage [51]	Wear and Tear g/km [22,35]	Wear and Tear Tonnes
Cars and 4-wheeled light vehicles	54,175,378	20,000	0.132	143,023
Motorised 2- and 3-wheelers	21,597,261	5200	0.007	786
Heavy trucks	2,488,680	51,500	1.068	136,882
Buses	888,393	73,500	0.204	13,320
Other	2,451,017	?	?	-
Total	81,600,729			294,011

**Table 13:** The amount of wear and tear of car tyres per capita per year (Number of capita as per July 2016 [42], Number of cars as per 2013 [52]).

	Number of Capita [42]	Number of Cars [52]	Total Emission from Tyres (tonnes/year)	Emission per Capita/year (kg)
The Netherlands	17,016,967	9,612,273	8834	0.52
Norway	5,265,158	3,671,885	7884	1.5
Sweden	9,880,604	5,755,952	13,238	1.3
Denmark	5,593,785	2,911,147	6721	1.2
Germany	80,722,792	52,391,000	92,594	1.1
United Kingdom	64,430,428	35,582,650	63,000	0.98
Italy	62,007,540	51,269,218	50,000	0.81
Japan	126,702,133	76,763,402	239,762	1.9
China	1,373,541,278	250,138,212	756,240	0.55
India	1,266,883,598	159,490,578	292,674	0.23
Australia	22,992,654	17,180,596	20,000	0.87
USA	323,995,528	265,043,362	1,524,740	4.7
Brazil	205,823,665	81,600,729	294,011	1.4
Total	3,564,856,130	1,011,411,004	3,369,698	0.95

**Table 14:** The amount of microplastics, including tyre wear and tear, passing the WWTP.

	Particles Passing the Sewers
Sweden	19.8%
Norway	5.3%
The Netherlands	28%

**Table 15:** The amount of wear and tear of car tyres compared to the total amount of plastics entering the oceans by land (pop. = population as per July 2016 [42]).

	A: Total Plastic into Oceans (tonnes/year) [4]	B: Tyre Wear and Tear into Oceans (tonnes/year)	% Tyre Wear and Tear of Total (B/(A + B))	Per Capita Tyre Wear and Tear Emission (kg/year/person)
The Netherlands (pop. 17,016,967)	27,700	261 (0.9%)	0.9%	0.015
Norway (pop. 5,265,158)	8400	3942 (31.9%)	31.9%	0.75

**Table 16:** Weight of Internal Combustion Engine (ICE)-cars compared to the electric version. The weight of the petrol car includes a half full tank.

Petrol Version	Weight [kg]	E-Version	Weight [kg]	Extra Weight E-Version
Volkswagen high Up! petrol	958	Volkswagen e-Up!	1114	16%
Volkswagen Golf 1.4 TSI	1205	Volkswagen E-Golf	1485	23%
Ford Focus	1380	Ford Focus Electric	1674	21%
Mercedes-Benz B 250	1465	Mercedes-Benz B 250 e	1725	18%
